# Patient-reported oral adverse events during cancer chemotherapy: longitudinal evaluation using patient-reported outcomes version of the common terminology criteria for adverse events (PRO-CTCAE) and concordance with clinician assessments

**DOI:** 10.1007/s00520-025-10126-3

**Published:** 2025-11-13

**Authors:** Yuki Sakai, Kouji Katsura, Yoshitomi Kanemitsu, Masaaki Kotake, Akira Toyama

**Affiliations:** 1 Department of Pharmacy, Niigata University Medical and Dental Hospital, 1-754 Asahimachi-Dori Chuoh-Ku, Niigata, 951-8520 Japan; 2 Department of Outpatient Cancer Chemotherapy Center, Niigata University Medical and Dental Hospital, Niigata, Japan; 3 Department of Oral Radiology, Niigata University Medical and Dental Hospital, Niigata, Japan

**Keywords:** Oral adverse events, Chemotherapy, Patient-reported outcomes, Multidisciplinary oral care, Outpatient cancer treatment, Concordance

## Abstract

**Purpose:**

This study aimed to (1) describe longitudinal trends in patient-reported oral adverse events using the patient-reported outcomes version of the common terminology criteria for adverse events (PRO-CTCAE) in patients receiving multidisciplinary oral care during outpatient cancer drug therapy, and (2) evaluate concordance between PRO-CTCAE and clinician-reported CTCAE scores.

**Methods:**

Conducted at the Outpatient Cancer Chemotherapy Center, Niigata University Medical and Dental Hospital (June–December 2023), oral adverse events were assessed using PRO-CTCAE and CTCAE at baseline and every 3 weeks up to 24 weeks. Concordance for dry mouth and oral mucositis was evaluated using paired same-day scores.

**Results:**

Among patients receiving multidisciplinary oral care, 85.2% experienced at least one oral adverse event, most commonly dry mouth. Based on PRO-CTCAE, symptoms rated as moderate or higher were reported in fewer than 10% of cases for most items. Notably, over half of patients reported persistent dry mouth of at least mild severity, and 20–30% of other symptoms also remained. These findings suggest that even lower-grade symptoms may persist and affect quality of life. Concordance between PRO-CTCAE and CTCAE was limited, with 31.1% of patients reporting greater dry mouth severity than clinicians documented.

**Conclusions:**

Patient-reported oral adverse events, as assessed by PRO-CTCAE, were frequently mild but persistent throughout outpatient chemotherapy, suggesting a cumulative impact on quality of life. Concordance between PRO-CTCAE and clinician-reported CTCAE was limited, indicating that clinician assessments may underestimate symptoms such as dry mouth. These findings underscore the need to integrate patient-reported outcomes into routine oral care.

## Introduction

Oral adverse events (AEs), such as mucositis, dry mouth, hoarseness, and taste changes, are common complications during cancer chemotherapy that can significantly impair quality of life (QOL) by affecting eating, speaking, and daily functioning [1]. Although clinician-reported assessments using the Common Terminology Criteria for Adverse Events (CTCAE) are standard for grading toxicity, evidence suggests they often underestimate the frequency and severity of subjective symptoms experienced by patients [2, 3]. To address this gap, the Patient-Reported Outcomes version of CTCAE (PRO-CTCAE) was developed [4–6], enabling more accurate capture of patient symptoms. The National Cancer Institute (NCI) indicated that using both CTCAE and PRO-CTCAE may improve recognition of mild AEs [7]. Although PRO-CTCAE has been increasingly utilized in oncology, longitudinal data specifically focusing on oral AEs during outpatient chemotherapy remain limited.

Our previous study demonstrated that patients receiving 5-fluorouracil (5FU)-, taxane-, and anthracycline-based regimens are particularly susceptible to oral complications and may benefit from specialized oral management [8]. Multidisciplinary oral care is widely recommended to prevent and manage these toxicities and thereby improve patient outcomes[9, 10]. Building on these findings, we developed a structured multidisciplinary oral care protocol for patients undergoing these regimens to facilitate consistent assessment and management of oral symptoms.

Moreover, direct comparisons between PRO-CTCAE and clinician-reported CTCAE for oral AEs remain limited, and the concordance between these assessment methods is not well understood [11, 12]. Given these considerations, a focused investigation was deemed necessary.

In this study, we aimed to characterize the frequency, severity, and longitudinal changes of patient-reported oral AEs using PRO-CTCAE among outpatients receiving multidisciplinary oral care. We also evaluated the concordance between PRO-CTCAE and clinician-reported CTCAE scores.

## Methods

### Study design

This was a retrospective observational study designed to evaluate longitudinal oral adverse events and symptom concordance among chemotherapy outpatients.

### Patients

This study was conducted at the Outpatient Cancer Chemotherapy Center of Niigata University Medical and Dental Hospital between June 1 and December 31, 2023. Patients were eligible for inclusion if they (1) had cancer, (2) were undergoing taxane-based and/or 5FU-based chemotherapy at the center, and (3) were between 18 and 100 years of age. Patients were excluded if they (1) never responded to the PRO-CTCAE, (2) were already receiving oral care from dental professionals, including dentists or dental hygienists.

Data on patient characteristics, including age, sex, smoking and alcohol history, activities of daily living (ADL), communication ability, medication management, and drug therapies, were extracted from electronic medical records.

### Ethical considerations

This study was conducted in accordance with the “Ethical Guidelines for Medical and Health Research Involving Human Subjects” and approved by the Ethics Committee of the Niigata University School of Medicine (approval number: 2023-0315).

### Oral AEs assessment

To assess oral AEs, the Japanese version 1.0 of PRO-CTCAE [13] and CTCAE version 5.0 were used. Patients reported seven oral AE symptom terms in the PRO-CTCAE: (1) dry mouth, (2) difficulty swallowing, (3) mouth/throat sores, (4) cracking at the corners of the mouth (cheilosis/cheilitis), (5) voice quality changes, (6) hoarseness, and (7) taste changes.

Dry mouth and oral mucositis were selected for clinician assessment using the CTCAE, as these AEs are well-defined and supported by established clinical criteria, ensuring greater inter-rater reliability and reproducibility. Although taste changes were frequently reported, their evaluation is highly subjective and relies on patient-reported outcomes rather than objective clinical assessment. Similarly, symptoms like hoarseness or voice quality changes are difficult to assess consistently in clinical practice. Therefore, we focused on the two most objectively evaluable and clinically relevant symptoms within CTCAE. In this study, PRO-CTCAE “Mouth/throat sores” was evaluated as equivalent to CTCAE “Oral mucositis,” as both terms reflect mucosal inflammation and associated symptoms [7, 14].

Patient- and clinician-reported scores were assessed on day 1 of each chemotherapy cycle. Chemotherapy was administered on various schedules (weekly, biweekly, or every 3–4 weeks) depending on the regimen, and patients were followed for up to 24 weeks from baseline. The number of cycles varied among patients based on their treatment protocol and clinical status. This data collection schedule reflected routine clinical practice.

### Statistical analysis

To evaluate concordance in the grading of dry mouth and oral mucositis between patients and clinicians, paired scores from the same day were analyzed. Concordance was defined as the same severity of adverse events reported by patients using the PRO-CTCAE (none, mild, moderate, severe, very severe) and by clinicians using the CTCAE (grades 0, 1, 2, 3, or 4) at a given time point [11], for a given time point. Weighted kappa value (k_w_) and its associated 95% confidence interval were calculated as a measure of concordance between assessment by patients and clinicians. This concordance is considered: poor, k_w_ < 0.2; fair, 0.21–0.40; moderate, 0.41–0.60; substantial, 0.61–0.80; and almost perfect, > 0.80.

All statistical analyses were performed using EZR ver.1.66 (Saitama Medical Center, Jichi Medical University, Saitama, Japan) [15], which is for R (The R Foundation for Statistical Computing, Vienna, Austria). It is a modified version of R commander designed to add statistical functions that are frequently used in biostatistics.

## Results

### Patient characteristics

**Fig. 1:**
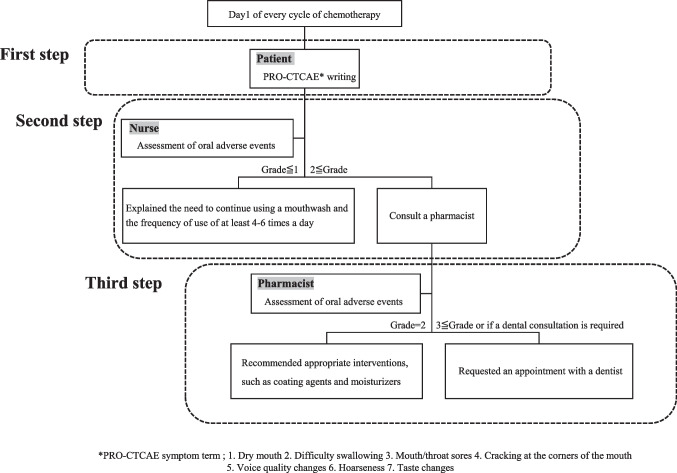
Multidisciplinary approach to oral care at the Outpatient Cancer Chemotherapy Center of Niigata University Medical and Dental Hospital

A total of 243 patients received oral care following the protocol shown in Fig. 1 at the Outpatient Cancer Chemotherapy Center of Niigata University Medical and Dental Hospital between June 1 and December 31, 2023. Of these, five patients did not receive taxane-based and/or 5FU-based chemotherapy, and one patient did not complete any PRO-CTCAE assessments. Therefore, 237 patients were included in the analysis. The mean age was 64 years (range, 23–84), and 112 (47.3%) were male. Most patients had normal physical and cognitive functions. Among the patients, 65.0% received taxane-based therapies, while 41.4% received 5-FU-based therapies. Six patients (2.5%) had a history of head and neck radiotherapy. Detailed demographic and clinical characteristics are summarized in Table 1.

**Table 1:** Patient characteristics

*N* = 237	*N*(%)
**Age(years)**	
Range	23–84
Median	64
**Sex**	
Male	112 (47.3%)
Female	125 (52.7%)
**Cancer type**	
Breast cancer	57 (24.1%)
Colon cancer	47 (19.8%)
Lung cancer	37 (15.6%)
Gastric cancer	24 (10.1%)
Pancreatic cancer	21 (8.9%)
Head and neck cancer	16 (6.8%)
Biliary tract cancer	14 (5.9%)
Prostate cancer	11 (4.6%)
Ovarian cancer	3 (1.3%)
Sarcoma	3 (1.3%)
Skin cancer	2 (0.8%)
Uterine cancer	1 (0.4%)
Neurocytoma	1 (0.4%)
**Smoking history**	
No-smoking	100 (42.2%)
Quit smoking(not active smoker)	109 (46.0%)
Smoking	19 (8.0%)
1 or 10 cigarettes a day	9
More than 10 cigarettes a day	10
Unknown	9 (3.8%)
**Alcohol history**	
No-drinking	153 (64.6%)
Habitual drinking	74 (31.2%)
Once a week	11
Three or four times a week	14
Every day	49
Unknown	10 (4.2%)
**ADL**	
Independence	223 (94.1%)
Assistance	10 (4.2%)
Unknown	4 (1.7%)
**Communication**	
No problem	223 (94.1%)
Hearing loss	5 (2.1%)
Others	1 (0.4%)
Unknown	8 (3.4%)
**Manage medicine**	
Self-management	194 (81.9%)
Family management	5 (2.1%)
Unknown	38 (16.0%)
**Chemotherapy**	
Fluorinated pyrimidines	98 (41.4%)
Taxanes	154 (65.0%)
**History of head and neck radiotherapy**	
Yes	6 (2.5%)
No	231 (97.5%)

### Oral care intervention by multidisciplinary team in routine care

Figure 1 illustrates the multidisciplinary oral care protocol for patients receiving outpatient chemotherapy. Based on our previous results, we targeted patients receiving 5FU- and taxane-based therapies [8].

All of nurses and pharmacists on the multidisciplinary team received a lecture and training on PRO-CTCAE version 1.0, oral care and the protocol from a well-trained dentist (K.K) before the intervention, and the intervention was carried out according to the protocol. Therefore, the nurses conducting the assessment and providing oral care instruction differed each cycle. However, the content of the assessment and the oral care instruction standardized. All procedures from first step to the third step were performed on the same day.

#### First step: Patient-rated oral adverse events

Patients completed the seven PRO-CTCAE items related to oral symptoms on day1 of each chemotherapy cycle at the Outpatient Cancer Chemotherapy Center of Niigata University Medical and Dental Hospital.

#### Second step: Nurses-rated oral adverse events and oral care education

The nurse reviewed PRO-CTCAE responses, assessed oral adverse events using the CTCAE version 5.0, and provided patient education on oral care on the same day. The nurse explained the need to continue using a mouthwash at least 4–6 times daily. Patients used mouthwash containing sodium azulene sulfonate or water. If the CTCAE grade was 2 or higher, the nurse consulted a pharmacist (Y.S or M.K).

#### Third step: Pharmacist-rated oral adverse events and suggested treatment drugs

The pharmacist evaluated oral symptoms and recommended appropriate interventions, such as coating agents for CTCAE-grade 2 oral mucositis and moisturizers for CTCAE-grade 2 dry mouth after mouth rinsing. Additionally, the pharmacist consulted with the medical oncologist regarding the prescription. If the CTCAE grade was 3 or higher, or if dental consultation was deemed necessary (e.g., when lubrication of the oral mucosa or dentist-prescribed materials were required), the pharmacist requested an appointment with a dentist.

### Frequency and severity of oral AEs assessed by PRO-CTCAE

**Fig. 2:**
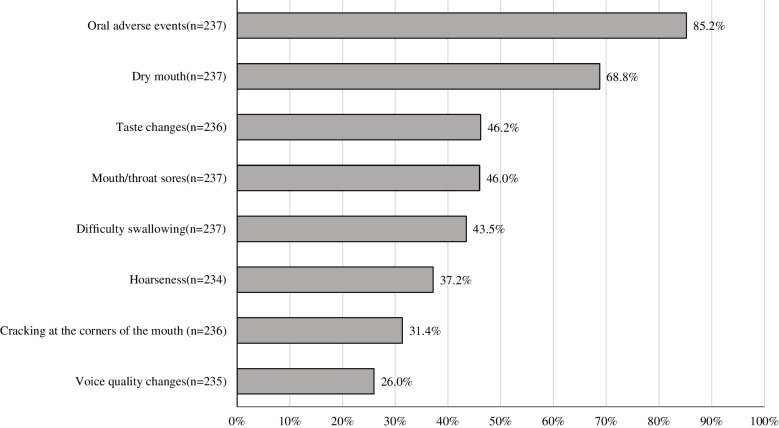
Proportion of patients who reported each symptom at least once during the 24-week PRO-CTCAE assessment period

**Fig. 3:**
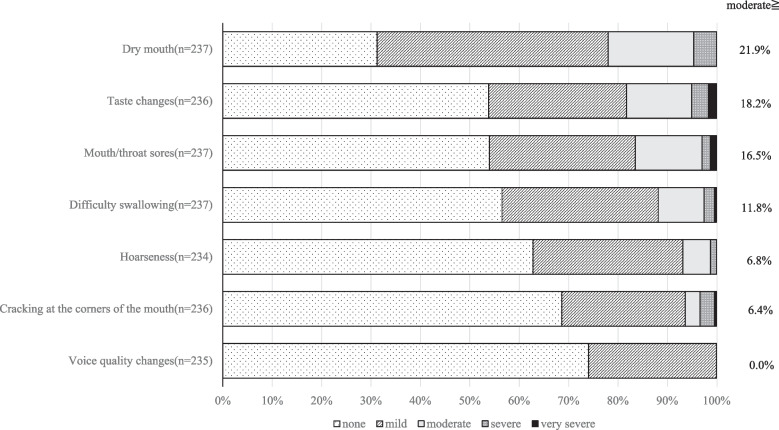
Proportion of patients by maximum PRO-CTCAE severity over 24 weeks. Percentages represent symptoms rated as moderate or higher

The frequency and severity of oral AEs assessed by PRO-CTCAE are shown in Figs. 2 and 3. 85.2% (202/237) of the patients experienced at least one symptom. Among these, dry mouth was the most frequently reported symptom (68.8%) (Fig. 2). In patients with a history of head and neck radiotherapy, the numbers were as follows: dry mouth (5/237), taste changes (1/236), mouth/throat sores (5/237), difficulty swallowing (5/237), hoarseness (3/234), cracking at the corners of the mouth(5/236), and voice quality change (3/235). Based on the highest PRO-CTCAE scores recorded during the study period, the proportion of patients reporting moderate or greater symptoms were as follows: dry mouth (21.9%), taste changes (18.2%), and mouth/throat sores (16.5%). In contrast, no patients reported changes in voice quality (0.0%) (Fig. 3).

### Longitudinal changes in PRO-CTCAE

**Fig. 4:**
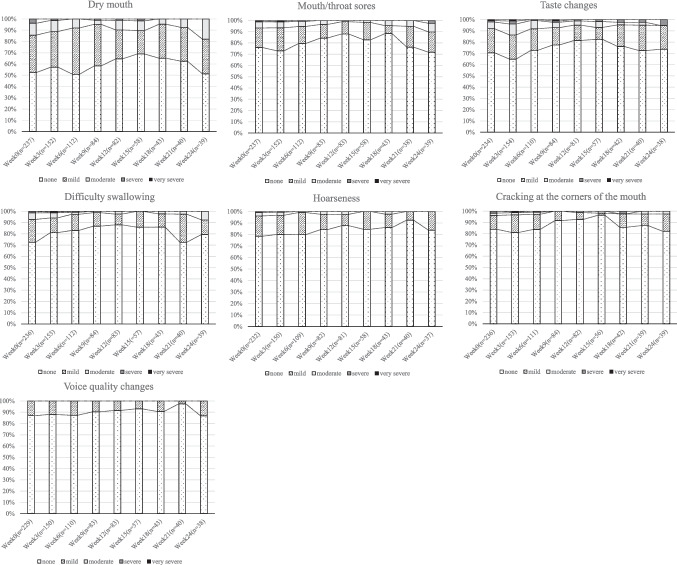
Longitudinal changes in PRO-CTCAE oral symptoms after initiation of multidisciplinary oral care

Figure 4 displays the distribution of oral symptom severity based on PRO-CTCAE grades at the initiation of multidisciplinary oral care and at subsequent 3-week intervals. The proportion of patients with moderate or greater AEs remained below 10% at nearly all time points, indicating that the severity of all symptoms was generally mild. However, the proportion of patients reporting any symptoms (as mild or greater) ranged from 30 to 50% for dry mouth, 20% to 35% for taste change, and 10% to 30% for mouth/throat sores, dysphagia, and hoarseness. For all symptoms, the proportion of patients reporting symptoms declined until approximately week 15, followed by a trend toward symptom exacerbation.

### Concordance between PRO-CTCAE and CTCAE

**Fig. 5:**
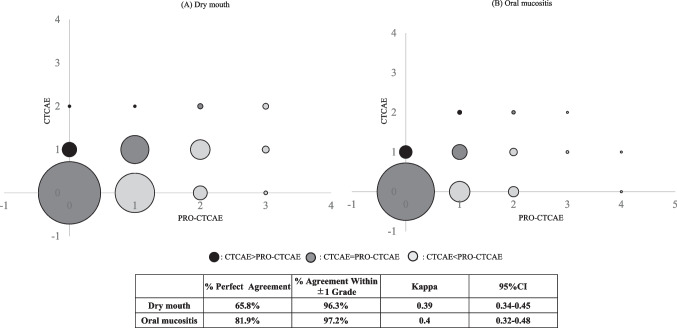
Agreement between patient and clinician reporting dry mouth (**A**) and oral mucositis (**B**). The number of agreements between patients and clinicians is illustrated by the size of the bubbles

To assess alignment between patient and clinician evaluations, we evaluated the agreement between PRO-CTCAE and CTCAE for dry mouth and oral mucositis. A total of 1066 assessment pairs for dry mouth and 1058 assessment pairs for oral mucositis were obtained. The agreement between patients and clinicians was fair for both dry mouth and oral mucositis (kw = 0.39, 95% CI = 0.34–0.45; kw = 0.4, 95% CI = 0.32–0.48). The distribution of PRO-CTCAE and CTCAE assessment pairs is shown in Fig. 5. The percentages of perfect agreement between patients and clinicians were 65.8% for dry mouth (agreement ± 1: 96.3%) and 81.9% for oral mucositis (agreement ± 1: 97.2%). Compared to clinicians, 31.1% (332/1066) of patients reported more severe dry mouth, while 14.0% (148/1058) reported more severe oral mucositis. Conversely, clinicians rated symptoms as more severe than patients in 3.1% of dry mouth cases and 4.1% of oral mucositis cases.

## Discussion

In this study, we developed multidisciplinary oral care and evaluated oral adverse events using PRO-CTCAE.

Three key findings emerged. First, 85.2% of patients experienced at least one oral adverse event, with dry mouth being the most frequently reported symptom; notably, 21.9% (52/237) reported dry mouth of moderate or greater severity. Second, although the severity of oral symptoms remained generally mild (≥ moderate < 10% throughout), a substantial proportion of patients reported persistent symptoms (≥ mild), such as dry mouth (30–50%), taste changes (20–35%), and oral mucositis, dysphagia, or hoarseness (10–30%) over the 24-week period, suggesting a prolonged impact on QOL. Third, the concordance between PRO-CTCAE and CTCAE for dry mouth and oral mucositis was limited, with 31.1% of patients reporting greater severity of dry mouth than assessed by clinicians.

The high incidence of oral adverse events (85.2%) among patients receiving 5-FU- and taxane-based regimens supports the validity of our screening approach and aligns with previous studies reporting strong associations between these agents and oral toxicities [1, 16, 17]. Targeting this population was therefore appropriate for establishing an effective referral system. Dry mouth was the most frequently reported adverse event (68.8%), consistent with prior findings showing rates of 56–69% in patients treated with 5-FU and 56% in those receiving taxanes [16]. These agents are known to reduce salivary secretion [18–20], likely contributing to the high frequency of reported dry mouth.

In this observational study using PRO-CTCAE, longitudinal monitoring of chemotherapy outpatients receiving multidisciplinary oral care showed that symptoms of ≥ moderate severity remained relatively infrequent, with most AEs persisting in the mild range. These findings suggest that ongoing multidisciplinary interventions may help prevent symptom escalation. However, the continued presence of ≥ mild symptoms—particularly dry mouth and taste changes—underscores the limitations of current care in fully alleviating these issues during treatment. Notably, after an initial decline in symptom prevalence, a rebound was observed beyond week 15, highlighting the need for sustained and extended intervention. Nonetheless, in the absence of a control group, we cannot definitively attribute these trends to the intervention rather than natural symptom fluctuations [21, 22].

The agreement between PRO-CTCAE and CTCAE was moderate (κ = 0.39–0.4), with 31.1% of patients reporting more severe dry mouth than clinicians, reflecting CTCAE’s limitations in capturing subjective symptoms [4, 23, 24]. Although perfect agreement was achieved in 65.8% of cases for dry mouth and 81.9% for oral mucositis, agreement within ± 1 grade was high (96.3% and 97.2%, respectively), suggesting that major discrepancies were infrequent. These findings highlight the structural differences between PRO-CTCAE and CTCAE: the former captures patients’ experiences, whereas the latter often relies on observable or functional changes, which may underestimate symptom burden [4, 25–31]. The relatively lower concordance for dry mouth likely stems from its subjective nature, lacking objective signs, unlike oral mucositis, which can be visually confirmed.

The tendency for patients to report greater symptom severity than clinicians further supports the integration of PROs into routine care to improve symptom recognition [32]. PRO-CTCAE and CTCAE provide complementary perspectives, and their combined use may enhance the sensitivity and accuracy of AE monitoring in clinical oncology [32].

This study has some limitations that should be considered when interpreting the findings. First, the study population was limited to patients receiving 5FU- and taxane-based chemotherapy, restricting the generalizability of findings to those undergoing different regimens. Second, dental examinations or microbiological tests were not performed, as dental evaluations were only conducted when CTCAE grades 2–4 were identified. Third, there may be variability in the frequency of oral care interventions, underscoring the need for a more standardized intervention protocol. Fourth, the follow-up period was limited to a maximum of 24 weeks, preventing assessment of long-term effects. Longer follow-up would be necessary to assess whether symptoms improve or worsen over time. Fifth, as this study lacks a control group, the effects of the multidisciplinary intervention cannot be conclusively determined. Further studies with a controlled design are needed to establish causality. Sixth, as the study was conducted at a single institution, potential biases related to patient characteristics should be considered. Finally, as this was a retrospective observational study, baseline and longitudinal assessments of QOL were not available. Although patient- and clinician-reported adverse events were evaluated using PRO-CTCAE and CTCAE, these measures do not fully reflect overall QOL. Future prospective studies incorporating baseline QOL assessments and longitudinal follow-up are warranted to determine whether such multidisciplinary oral care interventions improve patient outcomes and QOL. In addition, detailed information on salivary gland surgery and nutritional or weight changes was not consistently available in the medical records; therefore, subgroup analyses considering these clinical factors could not be performed. These variables may influence the duration and severity of oral symptoms, and future prospective studies should incorporate them to better identify patients who may benefit from targeted oral care interventions.

Our findings highlight the importance of regular monitoring by a multidisciplinary team for effective management of oral symptoms in cancer outpatients. Incorporating PRO-CTCAE into routine assessments may improve the detection of symptoms that are underrecognized by clinician-reported CTCAE alone. Given the limitations of recommending moisturizers and protective agents, future studies should evaluate the clinical impact of prescribing such interventions. Further research is also needed to assess the generalizability and long-term effectiveness of multidisciplinary oral care through multicenter studies, standardized intervention protocols, and extended follow-up durations.

## Conclusions

This study demonstrated that patient-reported oral AEs were frequently observed during outpatient chemotherapy, with dry mouth as the most common symptom. Longitudinal assessment using PRO-CTCAE showed that while the proportion of patients reporting symptoms fluctuated over time, oral AEs of at least mild severity persisted in 10% to 50% of patients throughout the 24-week observation period. The limited concordance between PRO-CTCAE and clinician-reported CTCAE—particularly for dry mouth—suggests that certain symptoms may be underrecognized in routine oncology practice. These findings support the integration of patient-reported outcome into multidisciplinary oral care to enhance symptom detection and management. Future studies should aim to standardize intervention protocols, evaluate the impact of prescription-based supportive care, and validate these findings in multicenter settings with longer follow-up.

## Data Availability

The datasets used and/or analysed during the current study are available from the corresponding author upon reasonable request **.**
